# Origin and Genetic Diversity of Diploid Parthenogenetic *Artemia* in Eurasia

**DOI:** 10.1371/journal.pone.0083348

**Published:** 2013-12-20

**Authors:** Marta Maccari, Francisco Amat, Africa Gómez

**Affiliations:** 1 School of Biological, Biomedical and Environmental Sciences, University of Hull, Hull, United Kingdom; 2 Instituto de Acuicultura de Torre de la Sal, Consejo Superior de Investigaciones Científicas, Castellón, Spain; Institute of Biochemistry and Biology, Germany

## Abstract

There is wide interest in understanding how genetic diversity is generated and maintained in parthenogenetic lineages, as it will help clarify the debate of the evolution and maintenance of sexual reproduction. There are three mechanisms that can be responsible for the generation of genetic diversity of parthenogenetic lineages: contagious parthenogenesis, repeated hybridization and microorganism infections (e.g. *Wolbachia*). Brine shrimps of the genus *Artemia* (Crustacea, Branchiopoda, Anostraca) are a good model system to investigate evolutionary transitions between reproductive systems as they include sexual species and lineages of obligate parthenogenetic populations of different ploidy level, which often co-occur. Diploid parthenogenetic lineages produce occasional fully functional rare males, interspecific hybridization is known to occur, but the mechanisms of origin of asexual lineages are not completely understood. Here we sequenced and analysed fragments of one mitochondrial and two nuclear genes from an extensive set of populations of diploid parthenogenetic *Artemia* and sexual species from Central and East Asia to investigate the evolutionary origin of diploid parthenogenetic *Artemia*, and geographic origin of the parental taxa. Our results indicate that there are at least two, possibly three independent and recent maternal origins of parthenogenetic lineages, related to *A. urmiana* and *Artemia* sp. from Kazakhstan, but that the nuclear genes are very closely related in all the sexual species and parthenogegetic lineages except for *A. sinica*, who presumable took no part on the origin of diploid parthenogenetic strains. Our data cannot rule out either hybridization between any of the very closely related Asiatic sexual species or rare events of contagious parthenogenesis via rare males as the contributing mechanisms to the generation of genetic diversity in diploid parthenogenetic *Artemia* lineages.

## Introduction

There is wide interest in understanding how genetic diversity is generated and maintained in parthenogenetic lineages, as it will help clarify the debate of the evolution and maintenance of sexual reproduction. Many asexual species are genetically diverse and this genetic diversity can to some extent ameliorate the lack of meiotic recombination [1], [2]. Several different genetic mechanisms underlie transitions from sexual reproduction to asexuality, and these mechanisms influence in turn the genetic diversity of parthenogenetic lineages and their success and persistence [3], [4]. However, some mechanisms of origin of parthenogenetic lineages can be recurrent, resulting in many, repeated non-independent but polyphyletic origins.

One mechanism for the polyphyletic origin of parthenogenetic lineages diversity is contagious parthenogenesis [3], in which parthenogenetically produced functional rare males mate with sexual females and transmit parthenogenesis to their offspring. Some parthenogenetic lineages produce functional rare males or invest in male function [3], [5], [6]. In the presence of sexual females of related lineages or species, rare males could produce fertile hybrid offspring which would inherit the parthenogenesis-inducing alleles. This mechanism has been best studied in the water flea *Daphnia pulex* [4], [7]–[9], but is also known to occur in the aphid *Myzus persicae* [10] and in the parasitoid wasp *Lisyphlebus fabarum* [11]. The genetic consequence of the spread of asexuality via contagious mechanism is the recurrent origin of new parthenogenetic clones, which will capture some genetic diversity of the maternal sexual species but also maintain some common genomic background from their parthenogenetic ancestor.

A second mechanism is the recurrent generation of multiple parthenogenetic lineages through recent hybridization between related sexual species [3]. Parthenogenesis can result from hybridization between two co-occurring sexual species in vertebrates [12]–[14] and in invertebrates [3], [15], [16]. The repeated origin of hybrid asexuals might generate complex patterns of relationships between the parthenogenetic lineages [17].

A third mechanism of polyphyletic origin is through infection by vertically inherited microorganisms, such as *Wolbachia* [3]. Microorganisms associated with parthenogenesis can alter the reproduction of their host to favour their persistence in populations, for example by feminizing or killing males or inducing parthenogenesis [2], [18].

If parthenogenetic lineages arise repeatedly trough these mechanisms or a combination of them, their genetic diversity may be comparable to those of sexual populations [1], [19], [20]. Such repeated transitions between sexual and asexual lineages can generate many related but highly diverse asexual lineages which can potentially lead to confounding estimates of genetic diversity of parthenogenetic lineages, and conclusions of ancient asexuality [16].

Brine shrimps of the genus *Artemia* (Crustacea, Branchiopoda, Anostraca) are a good model system to investigate evolutionary transitions between reproductive systems as they include sexual species and lineages of obligate parthenogenetic populations of different ploidy level [21]. Parthenogenetic populations are found only in the Old World, where they co-occur with various sexual species, including *A. salina* (Linnaeus 1758) in the Mediterranean region and South Africa [22], *A. urmiana* (Günther 1899) in and around lake Urmia (Iran) and Crimean salt lakes [23], *A. sinica* in Central and Northern China [24], *A. tibetiana* in the Tibetan plateau [25], [26], and likely with a yet undescribed sexual species in Kazakhstan [27], [28]. *Artemia* species differ in genetic, morphometric, morphological, life history traits [23], [28], and show reproductive isolation, although this is weaker between Asiatic species [25].

Parthenogenetic diploid *Artemia* populations are automictic and most populations produce fully functional males in low proportions (from 1 to 17 per thousand individuals)[29]. These so called rare males can produce fertile offspring when mating with females of sexual Asiatic species [29]. Assessments of the mitochondrial genetic diversity of Mediterranean parthenogenetic *Artemia* populations suggested that there were at least two maternal origins of diploid parthenogenesis from a group of closely related Central Asiatic sexual species [30]: one of the mitochondrial lineages – largely responsible for the recent expansion of diploid parthenogenetic *Artemia* in the Mediterranean – is closely related to those of a sexual undescribed species from Kazakhstan, and the other, rarer lineage, which is closely related to haplotypes of Iranian *A. urmiana*. The occurrence of two diploid parthenogenetic lineages, and the origin of triploid strains from the common parthenogenetic lineage was also supported by a study of microsatellite and mtDNA sequence diversity of parthenogenetic populations [31]. Nuclear gene sequence variation such as ITS1 [32], also indicated that there were multiple origins of parthenogenesis amongst the sexual species from Asia including *A. urmiana*, *A. tibetiana* and *A. sinica*, but as the ploidy of the samples was not identified, conclusions could not be drawn regarding the origin of diploid parthenogenetic *Artemia*. However, *A. salina* and the two American species, are only distantly related to parthenogenetic lineages [32].

Although diploid parthenogenetic *Artemia* can be identified by their morphology, a genetic marker to characterise would be very useful. In this respect, a study by Manaffar et al. [33] revealed that the digestion of the fragment of exon-7 of Na^+^/K^+^ ATPase by *Tru1I* restriction enzyme showed a polymorphism that allowed discriminating between sexual species and parthenogenetic populations. The sexuals resulted to be homozygote whereas the parthenogens were heterozygote in this position.

Little is known about the mechanisms of origin of parthenogenetic lineages from the ancestral sexual condition, although the possibility of an infectious origin of parthenogenetic *Artemia* lineages through *Wolbachia* parasites has been ruled out [34]. Given the functionality of rare males when crossed with Asiatic sexual females, Maccari et al. [29] suggested that they may have an evolutionary role through genetic exchange between parthenogenetic lineages and Asiatic related sexual species. Another possibility would be a hybrid origin between two related sexual species which could give rise to parthenogenetic lineages, especially given the evidence for interspecific hybridization in *Artemia* in natural populations [35] and in the laboratory [25]. The limited analysis of Asiatic diploid parthenogenetic populations, where the coexistence with closely related sexual species is more likely, has also hampered our understanding of the origin of parthenogenetic lineages.

Here we obtained and analysed sequences from one mitochondrial and two nuclear genes (including the putatively diagnostic marker Na^+^/K^+^ ATPase) from an extensive set of populations of diploid parthenogenetic *Artemia* and sexual species with emphasis on Central and East Asia in order to gain insights into the evolutionary origin of diploid parthenogenetic *Artemia*, its mode of origin and geographic origin of the parental taxa.

## Materials and Methods

### Samples

**Figure 1:**
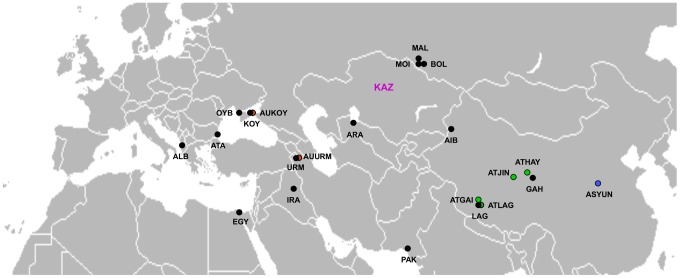
Map of geographic distribution of *Artemia* populations sampled. Black circles represent diploid parthenogenetic populations and coloured ones sexual species. Note that due to its unknown locality, *Artemia* sp. Kazakhstan is represented without circle. See Table 1 for population codes.

Cyst samples from 15 Eurasian populations of diploid parthenogenetic *Artemia* (from here onwards, we will use ‘parthenogenetic *Artemia*’ or ‘parthenogens’ to refer to diploid parthenogenetic *Artemia* for simplicity) were obtained from the cyst bank collection of the Instituto de Acuicultura de Torre de la Sal (IATS-CSIC) (Figure 1). Laboratory populations were reared from these cyst samples. We assessed the reproductive mode of each population using a sex ratio criterion [29] and whenever the original cyst samples contained an additional sexual species (see Table 1), we obtained pure laboratory parthenogenetic populations using morphometric methods (for culture conditions and other details see [29]). Cyst samples from Asiatic sexual species were also obtained from the same cyst bank collection, including *A*. *urmiana* from Urmia lake and from Koyashskoe lake, *A. tibetiana* from four lakes of the Tibetan plateau (Lagkor Co, Gaize, Hayan, Jingyu), an undescribed sexual *Artemia* population from Kazakhstan (originally Artemia Reference Center code - ARC 1039, unknown locality) and *A. sinica* from Yuncheng (China) (Figure 1).

**Table 1:** Detailed information on *Artemia* samples: population name, population codes, location details and additional co-occurring species found in the sample.

	Population	Codes	Coordinates	Other species
***Diploid parthenogens***	Narte saltern, Albania	ALB	40°34′46″N-19°28′16″E	
	Atanasovko Lake,Bulgaria	ATA	42°34′25″N-27°28′09″E	
	Oybuskoye Lake, Ukraine	OYB	45°16′15″N-33°04′40″E	
	Koyashskoe Lake,Ukraine	KOY	45°02′09″N-36°12′00″E	*A. urmiana*
	Alexandria saltern,Egypt	EGY	31°04′13″N-29°46′57″E	
	Bagdad saltern, Iraq	IRA	33°20′19″N-44°29′32″E	
	Urmia Lake, Iran	URM	37°20′00″N-45°40′00″E	*A. urmiana*
	Aral Sea, Uzbekistan	ARA	45°00′00″N-59°56′00″E	
	Maloje Jarovoe Lake, W.Altai	MAL	52°47′31″N-79°33′39″E	
	Bolshoe Jarovoe Lake, W.Altai	BOL	52°50′N-79°45′E	
	Moimishanskoe Lake, W.Altai	MOI	52°50′N-79°45′E	
	Korangi Creek saltern, Pakistan	PAK	24°47′25″N-67°09′33″E	
	Aibi Lake, China	AIB	44°45′42″N-82°51′54″E	
	Lagkor Co Lake, Tibet	LAG	32°03′N-84°13′E	*A. tibetiana*
	Gahai Lake, China	GAH	36°58′18″N-98°09′53″E	
***Sexuals***				
*A. urmiana*	Koyashskoe Lake, Ukraine	AUKOY	45°02′09″N-36°12′00″E	*diploid parthenogenetic*
*A. urmiana*	Urmia Lake, Iran	AUURM	37°20′00″N-45°40′00″E	*diploid parthenogenetic*
*Kazakhstan sp.*	*unknown, Kazakhstan*	KAZ	?	
*A. tibetiana*	Lagkor Co Lake, Tibet	ATLAG	32°03′N-84°13′E	*diploid parthenogenetic*
*A. tibetiana*	Gaize Lake, Tibet	ATGAI	32°20′N- 84°10′E	
*A. tibetiana*	Jingyu Lake, Tibet	ATJIN	36°03′N-89°09′E	
*A. tibetiana*	Hayan Lake, Tibet	ATHAY	36°03′N-100°11′E	
*A. sinica*	Yuncheng saltern, China	ASYUN	35°00′N-111°00′E	

### DNA isolation, polymerase chain reaction, and sequencing

Total DNA was extracted from cysts using a modified HotSHOT protocol [36]. We amplified fragments of one mitochondrial (cytochrome c oxidase subunit I, COI) and two nuclear genes (internal transcribed spacer 1, ITS1, and Na^+^/K^+^ ATPase).

The COI fragment was amplified using the primers HCO2198 and LCOI490 [37]. PCR was carried out in a total volume of 50 µl containing 5 µl of template DNA, 0.2 mM of each nucleotide, 0.2 µM of each primer, 0.05 U of *Taq* polymerase (Bioline) and 10×Bioline buffer (producing a MgCl_2_ final concentration of 2 mM). The cycling profile consisted of one cycle of 3 min at 95°C, followed by 40 cycles of 15 s at 95°C, 20 s at 50°C, and 30 s at 72°C, with a final step of 5 min at 72°C.

PCR of the ITS1 region was performed using primers PTF and PTR [38] in a total volume of 30 µl consisting of 3 µl of template DNA, 0.2 mM of each nucleotide, 0.2 µM of each primer, 0.03 U of *Taq* polymerase (Bioline) and 10×Bioline buffer (producing a MgCl_2_ final concentration of 1.5 mM) using the following conditions: a cycle of 3 min at 95°C, followed by 35 cycles of 60 s at 95°C, 50 s at 59°C, and 90 s at 72°C, and a final step of 7 min at 72°C.

A fragment of 280-bp, representing exon-7 of Na^+^/K^+^ ATPase, was amplified using the primers designed by [33]. PCR was performed in a total volume of 20 µl, containing 3 µl of template DNA, 0.2 mM of each nucleotide, 0.2 µM of each primer, 0.02 U of T*aq* polymerase (Bioline) and 10×Bioline buffer (producing a MgCl_2_ final concentration of 2 mM) using the following program: 94°C for 2 min, 32 cycles at 94°C for 25 s followed by 56°C for 25 s and 72°C for 1 min, and a final extension at 72°for 3 min.

All amplifications were performed on a Verity 96 well thermal cycler (Applied Biosystems). PCR products were purified and sequenced by Macrogen Europe Inc. (Amsterdam, The Netherlands). The electrophoregrams were checked by eye using CodonCode Aligner v. 3.5 (CodonCode Corporation, Dedham, MA). COI and ITS1 sequences generated were deposited in GenBank (for Accession Numbers see Tables 2 and 3) and all alignments are available in Dryad (http://doi.org/10.5061/dryad.kd0k4).

**Table 2:** COI samples and haplotypes: sample size; number of haplotypes per population; πJC, corrected nucleotide diversity; *Hd*, gene diversity.

Population code	Sample size	Number of haplotypes	Haplotypes and sample size	*πJC*	*Hd*	*Acc.Num*
***Diploid parthenogens***						
URM	20	2	APD02(17), APD05(3)	0.0009	0.2684	KF707710-19, KF707765-74
KOY	15	1	APD02(15)	0.0000	0.0000	KF707700-09, KF707805-09
ATA	12	3	APD02(10), APD07(1), APD12(1)	0.0071	0.3182	KF707720-26, KF707800-04
IRA	19	1	APD02(19)	0.0000	0.0000	KF707727-45
EGY	5	2	APD02(3), APD05(2)	0.0020	0.6000	KF707785-89
ALB	10	2	APD02(2), APD05(8)	0.0012	0.3556	KF707790-99
PAK	10	1	APD02(10)	0.0000	0.0000	KF707775-84
OYB	10	2	APD10(3), APD08(7)	0.0008	0.4667	KF707810-19
ARA	6	4	APD02(2), APD11(2), APD13(1),APD14(1)	0.0021	0.8667	KF707820-25
MAL	10	3	APD02(3), APD15(5),APD16(2)	0.0015	0.6889	KF707826-35
BOL	9	3	APD02(7), APD15(1),APD16(1)	0.0007	0.4167	KF707836-44
MOI	10	3	APD02(2),APD18(7), APD19(1)	0.0026	0.5111	KF707865-74
AIB	9	3	APD02(5), APD09(1),APD10(3)	0.0136	0.6389	KF707746-54
GAH	10	1	APD11(10)	0.0000	0.0000	KF707755-64
LAG	10	3	APD02(4), APD05(1),APD17(5)	0.0145	0.6444	KF707845-54
***Sexuals***						
KAZ	10	4	KAZSEX06(2), KAZSEX05(2), KAZSEX03(4), KAZSEX08(2)	0.0038	0.8000	KF707671-80
AUURM	20	12	AUURM01(1), AUURM02(1), AUURM03(1), AUURM04(7), AUURM05(1), AUURM06(1), AUURM07(1), AUURM08(1), AUURM09(1), AUURM10(2), AUURM11(2), AUURM12(1)	0.0074	0.8790	KF707681-90, KF707875-84
AUKOY	9	2	AUKOY01(5),AUKOY02(4)	0.0027	0.5556	KF707691-99
ATLAG	20	4	AT01(17), AT08(1), AT09(1),AT10(1)	0.0007	0.2842	KF707855-64, KF707919-28
ATGAI	5	1	AT01(5)	0.0000	0.0000	KF707895-99
ATHAY	9	4	AT02(3),AT03(4), AT04(1), AT05(1)	0.0036	0.7500	KF707900-08
ATJIN	10	3	AT05(4), AT06(1), AT06(5)	0.0015	0.6444	KF707909-18
ASYUN	10	2	AS01(6), AS02(4)	0.0017	0.5333	KF707885-90

**Table 3:** Nuclear loci summary of polymorphic sites in each *Artemia* population. A dash means that heterozygote individuals were found, a forward slash indicate that the position is polymorphic in the population, with both homozygote and heterozygotes found.

		ITS					NA+/K+ ATPase						
		Sample size	522bp	721bp	695bp	*Acc. Num.*	Sample size	26bp	56bp	80bp	95bp	140bp	152bp
***Diploid parthenogens***	**ALB**	2	C	C	T	KF736274,75	2	C	T	T	A	G-T	T
	**ATA**	2	A	C	T	KF736258,59	2	C	T	T	A	G-T	T
	**OYB**	2	A	C	T	KF736276,77	3	C	T	T	A	G-T	T
	**KOY**	2	C-A/A	C	T	KF736255-57	2	C	T-C	T	A	G-T	T
	**EGY**	2	C	C	T-A	KF736266-69	2	C	T	T	A	G-T	T
	**IRA**	2	A	C	T	KF736264,65	4	C	T	T	A	G-T	T
	**URM**	2	C	C	T	KF736253,54	2	C	T-C/T	T-A/T	A	G-T	T
	**ARA**	2	C/A	C	T	KF736278,79	2	C	T-C	T	A	G-T	T
	**MAL**	2	C	C	T	KF736280,81	2	C	T-C	T	A	G-T	T
	**BOL**	2	C	C	T	KF736282,83	2	C	T	T	A	G-T	T
	**MOI**	2	C	C	T	KF736284,85	3	C	T-C	T-A	A	T/G-T	T
	**PAK**	2	A	C-T	T	KF736270-73	2	C	T	T	A	G-T	T
	**AIB**	2	C	C	T	KF736260,61	2	C	T	T	A	G-T	T
	**LAG**	2	C-A	C	T	KF736286-89	4	C	T	T	A	G-T	T
	**GAH**	2	C	C	T	KF736262,63	2	C	T-C	T-A	T-A	T	T
													
***Sexuals***	**AUKOY**	2	C	C	T	KF736251,52	5	C	T	T	A	T	T
	**AUURM**	2	C	C	T	KF736249,50	4	C	T	T	A	T	T
	**KAZ**	2	C	C	T	KF736247,48	6	C	T-C	T-A	A	T	T
	**ATLAG**	2	C	C	T	KF736290,91	3	C	T	T	A	T	T
	**ATGAI**	2	C	C	T	KF736294,95	4	C	T	T	A	T	T
	**ATJIN**	2	C	C	T	KF736291,92	3	C	T	T	A	T	T
	**ASYUN**	2	C	T	T	KF736296,97	2	T	T	T	A	T	C-T

### Sequence alignment and phylogenetic analyses

The COI fragment was sequenced in 258 individuals, 165 of which were diploid parthenogens (see Table 2). For the nuclear markers we sequenced a subset of these individuals, 44 for the ITS1 region (two for each population sampled) and 63 for the Na^+^/K^+^ ATPase fragment (Table 3).

To the COI marker alignment we also added 55 published available sequences from GenBank (parthenogenetic rare males and females KC193638-KC193677, parthenogenetic haplotypes DQ426824-DQ426826, haplotypes from parthenogenetic populations and from *Artemia sp.* Kazakhstan GU591380-GU591389 and *A.tibetiana* EF615588-89). Sequences were aligned using ClustalW in MEGA5 [39] using the default settings and checked by eye. The number of polymorphic and parsimony informative sites was computed with MEGA5. Patterns of nucleotide diversity, synonymous and non-synonymous substitutions, population haplotype and nucleotide diversity were computed using DnaSP5 [40].

Before phylogenetic reconstruction, sequences were collapsed into haplotypes using FaBox v.1.40 [41]. For both COI and ITS1 markers, phylogenetic analysis was implemented using Maximum Likelihood (ML) approaches in MEGA5 and Bayesian approaches in MrBayes v 3.2.2 [42] on the Cipres Science Gateway portal [43]. We estimated the best-scoring ML tree using the model selected by the inbuilt model generator in MEGA5. The robustness of the branches was assessed with 1000 bootstrap pseudo-replicates. For Bayesian analysis we used the default parameters on the Cipres gateway. In two simultaneous runs, four Markov chains (one cold and three heated) were started from a random tree and run for 1,000,000 generations with sampling frequency every 100 generations. The first 2500 trees were discarded as burn-in.

In addition, we constructed a statistical parsimony haplotype network for COI using TCS v. 1.21 [44] to visualize the genealogical relationships between the mitochondrial haplotypes. For this analysis we used all the COI sequences generated here, two *A. tibetiana* sequences from GenBank (EF615587-8), the sequences from Maccari et al. [29] and Muñoz et al. [30]. For sequences from the latter paper, including Mediterranean populations of diploid parthenogenetic *Artemia*, we reconstructed the sequence of each individual from the paper haplotype information.

## Results

### Cytochrome oxidase subunit I

The sequence alignment was trimmed to 614 bp long, with all the 313 sequences of the same length. No insertions, deletions or stop codons were present. The COI alignment consisted of 143 variable sites and 133 parsimony informative sites with a total of 144 synonymous and 10 nonsynonymous substitutions.

The sequences generated here collapsed into 45 haplotypes (see Table 2). No haplotype was shared between parthenogens and sexuals, despite both parthenogens and sexuals coexisting in three of the sampled populations. Diploid parthenogenetic populations had a total of 15 haplotypes, 11 of them newly found in this study. APD02, the most common and widespread haplotype, was found in 99 individuals from 13 out of the 15 diploid parthenogenetic populations sampled. The next most common haplotype, APD05 was found in four populations (URM, EGY, ALB and LAG), APD10 in two populations (OYB and AIB), as APD11 (ARA and GAH). Haplotypes APD15, APD16 were found in both populations from the Altai (MAL and BOL). The remaining nine haplotypes were found in single populations.

The sexual populations sequenced here had 30 COI haplotypes. We found four exclusive haplotypes in the undescribed sexual species from Kazahkstan, 12 in *A. urmiana* from Urmia Lake, and two in *A. urmiana* from Koyashskoe Lake, with no shared haplotypes between these *A. urmiana* populations. The populations of *A. tibetiana* had 11 haplotypes. The population of *A. sinica* was characterized by two haplotypes. The highest haplotype diversity (*Hd*) was found in *A. urmiana* from lake Urmia (0.88) and in the parthenogenetic population from Aral Sea (0.87) (Table 2). Populations from Koyashskoe Lake, Bagdad saltern, Korangi Creek saltern and Gahai Lake amongst the parthenogens and *A. tibetiana* from Gaize Lake among the sexuals were characterized by a single haplotype.

The nucleotide diversity values (π-values) ranged from 0.0000 to 0.0145 (Table 2). The highest value was found in two parthenogenetic populations from Lagkor Co and Aibi Lake, but the sexual populations from Urmia Lake, Kazakhstan and Hayan Lake and the parthenogenetic population from Atanosovko Lake also showed high π-values compared with the rest of the populations.

**Figure 2:**
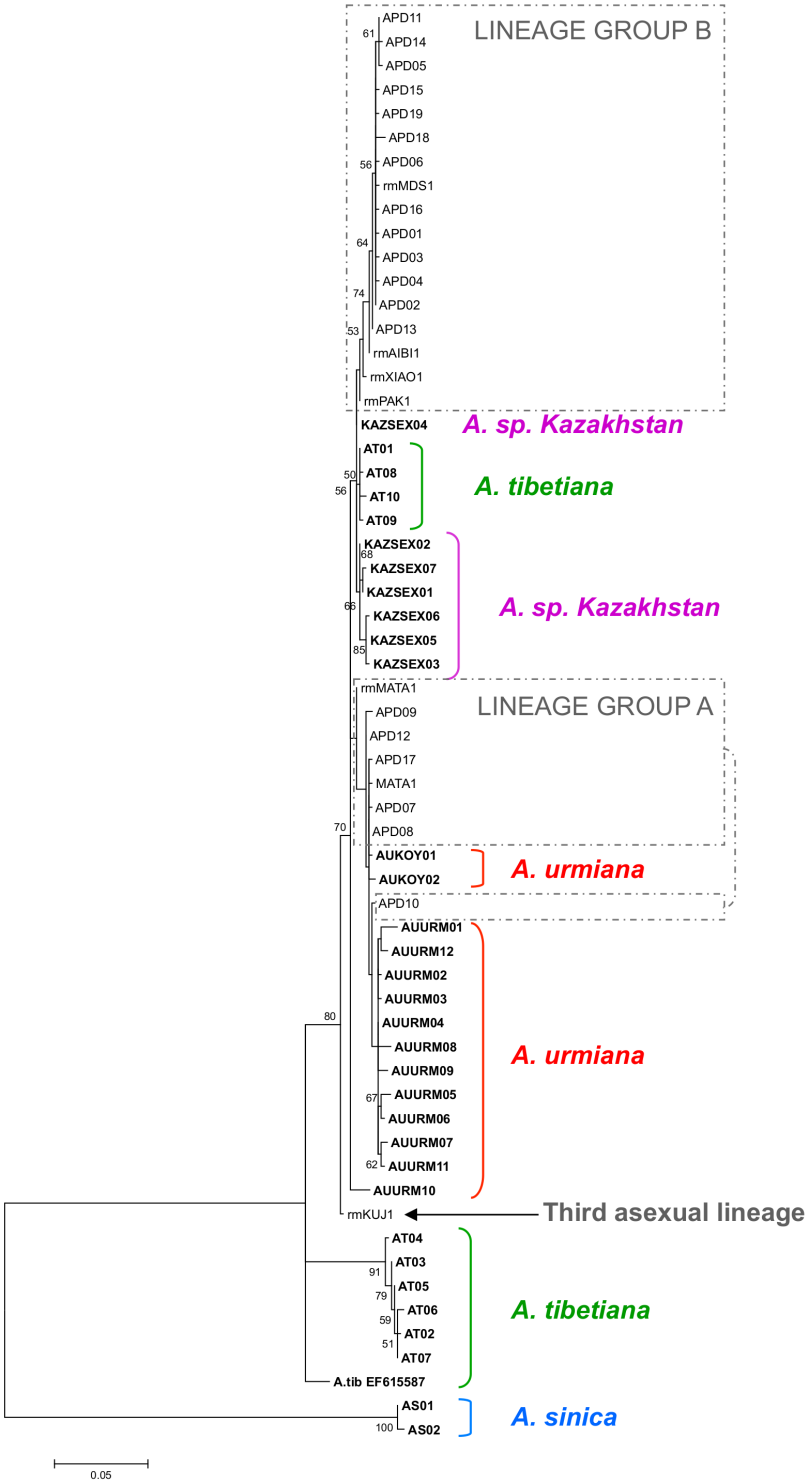
Maximum Likelihood (ML) phylogenetic tree of diploid parthenogenetic *Artemia* and Asiatic sexual species based on COI haplotypes. Sequence evolution is based on the T92 + G model. One thousand pseudoreplications of bootstrapping were used. For haplotypes from GenBank, the code for each haplotype shown corresponds to the code for the first individual in the alignment with that haplotype (see text, Table 2 and Figure 4 for the individuals included in each haplotype). Sexual species are shown in bold. Rare males are noted by rm followed by the population code as reported en GenBank.

**Figure 3:**
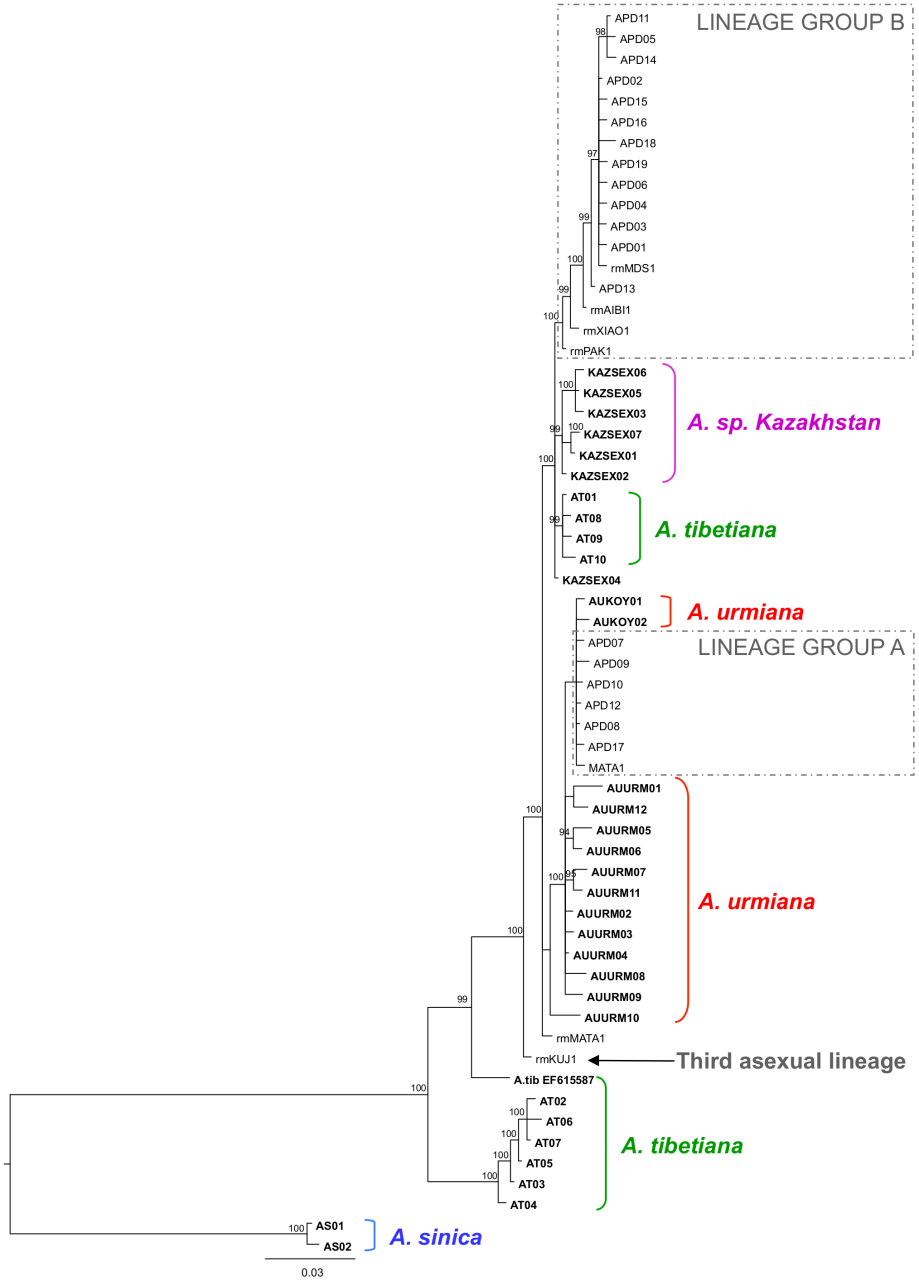
Bayesian inference of phylogenetic relationships of diploid parthenogenetic *Artemia* and Asiatic sexual species based on COI haplotypes. Support values higher than 0.90 are shown. For haplotypes from GenBank, the code for each haplotype shown corresponds to the code for the first individual in the alignment with that haplotype (see text, Table 2 and Figure 4 for the individuals included in each haplotype). Sexual species are shown in bold. Rare males are noted by rm followed by the population code as reported en GenBank.

The ML tree (Figure 2) was obtained using the Tamura-3 parameter (T92) plus gamma model, the one selected by the inbuilt model generator in MEGA5. The tree showed that all diploid parthenogenetic *Artemia* haplotypes, plus the haplotypes of *A*. *urmiana* populations, *Artemia* sp. Kazakhstan and the haplotypes of *A. tibetiana* from Lagkor Co and Gaize Lake formed a highly supported monophyletic lineage. A group of diploid parthenogenetic *Artemia* haplotypes formed a polyphyletic, not well supported assemblage amongst haplotypes from both *A. urmiana* populations (lineage group A). A second group of haplotypes, including the most common APD02 haplotype, formed a monophyletic, but not highly supported lineage, closely related to *Artemia* sp. Kazahkstan and to the lineage of *A. tibetiana* (which we called lineage group B). The haplotype from Kujalnik (rmKUJ1), obtained in two rare males [29] formed a well supported sister branch to those containing all other parthenogenetic. The mtDNA lineages of the other two *A. tibetiana* populations (Hayan and Jingyu Lake) and *A. sinica* were only distantly related to those of diploid parthenogenetic *Artemia*. The Bayesian consensus tree (Figure 3) showed a similar topology, although it resolves the relationships of two *A. tibetiana* lineages. *A. tibetiana* from GenBank (EF615587) forms a highly supported branch with all diploid parthenogens, *A. urmiana*, *Artemia* sp. Kazakhstan and the haplotypes of *A. tibetiana* from Lagkor Co and Gaize Lake. Lineage group A, with the exception of rmMATA1, together with all *A. urmiana* haplotypes forms a well supported lineage. Lineage group B forms a well supported monophyletic lineage and its relationship with *Artemia* sp. Kazakhstan and the haplotypes of *A. tibetiana* from Lagkor Co and Gaize Lake was also highly supported. Further differences with the ML analysis are represented by the position of AURM010, which in the Bayesian analysis falls at the base of the rest of *A. urmiana* haplotypes and Lineage group A, and by the position of rmMATA1 which forms a polytomy more basal in the tree, instead of belonging to lineage group A.

**Figure 4:**
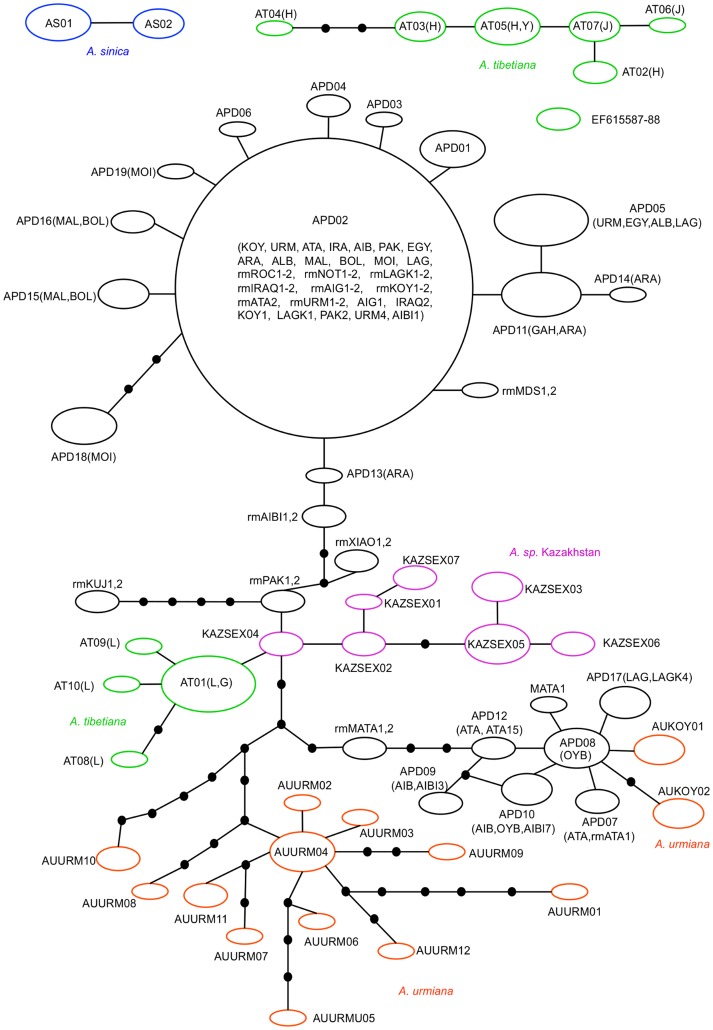
Statistical Parsimony networks showing the nested relationships of diploid parthenogenetic *Artemia* haplotypes and Asiatic sexual species. Black circles represent diploid parthenogenetic *Artemia* haplotypes and coloured circles represent Asiatic sexual species. Circle diameter is proportional to the relative haplotype frequency. Connecting lines indicate single substitutions and small black circles represent putative missing haplotypes. The haplotypes codes correspond to those listed in Table 2 or those from GenBank. Rare males are noted by rm followed by the population code as reported en GenBank.

The statistical parsimony network shows the relationship between the mitochondrial haplotypes of parthenogenetic and related sexual species more clearly (Figure 4). There were four unlinked networks. The two haplotypes from *A. sinica* formed a network, the two *A. tibetiana* populations from Hayan and Jinyu Lake resulted in a second haplotype network, and the two *A. tibetiana* sequences from GenBank (EF615587-88) formed a third network. The remaining haplotypes including all diploid parthenogenetic samples, *A. urmiana*, *Artemia* sp. from Kazakhstan and the *A. tibetiana* populations of Lagkor Co and Gaize Lake were joined in a single network. Haplotypes of diploid parthenogenetic *Artemia* formed three distinct mitochondrial lineage groups as in the phylogenetic reconstructions. Lineage group A, with eight haplotypes, is nested within the diversity of *A. urmiana* haplotypes and most closely related to haplotypes from Koyashkoe Lake population. This is a relatively rare parthenogenetic lineage, but found at very geographically widespread populations (Atanosovsko Lake, Oybuskoye Lake, Lagkor Co Lake, la Mata Lagoon and Aibi Lake parthenogenetic populations). Lineage group B is more common and widespread, and is formed by the common haplotype APD02 and a number of closely related ones forming a star-like network. Lineage group B is closely related to haplotypes from *A. tibetiana* from Lagkor Co and Gaize Lake (AT01, AT08, AT09 and AT10) and *Artemia* sp. from Kazakhstan (KAZSEX01-07), which are also closely related between them. There is no geographic association of the two lineages with a well-defined region because both diploid parthenogenetic haplotype lineage groups coexist in Atanosovsko Lake (ATA), Aibi Lake (AIB) and Lagkor Co Lake (LAG) populations. Some haplotypes found exclusively in rare males from diploid parthenogenetic populations of diverse origins (rmPAK from Korangi Creek in Pakistan; rmXIAO from Xiaotan in China; rmMATA from La Mata in Spain) appeared in the center of the network, and were more closely related to haplotypes of sexual populations. The haplotype from rare males of Kujalnik (rmKUJ from Kujalnik in Ukraine) formed a separate branch to the rest, and would be a third group of parthenogenetic lineages.

### ITS-1

The ITS1 sequences, excluding gaps in the alignment, ranged from 991 (*A. tibetiana*, *Artemia* sp. from Kazakhstan, *A. urmiana* from Koyashskoe lake and all the parthenogens) to 1000 bp (*A. sinica*), including the sequences of *A. urmiana* from Urmia lake which have a variable length (994–999 bp). The final ITS1 alignment was 1002 bp long, with 34 variable sites and 28 parsimony informative sites and collapsed into 14 haplotypes. Evidence of heterozygosity was found in 5 parthenogenetic populations and allele identification in these was straightforward (Table 3).

**Figure 5:**
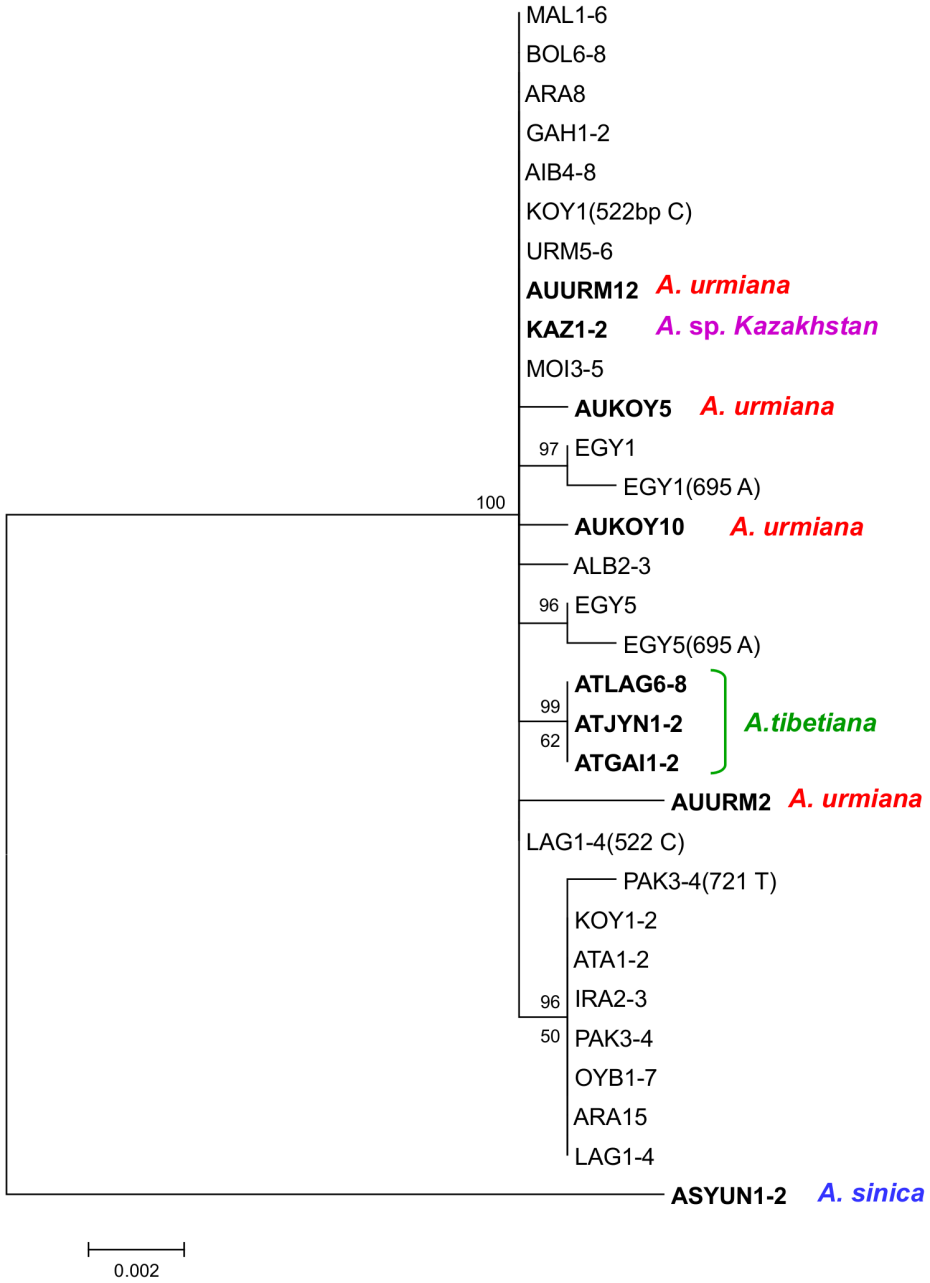
Phylogenetic relationships of diploid parthenogenetic *Artemia* and Asiatic sexual species based on ITS-1 sequences. The topology inferred by Maximum Likelihood (ML) method using HKY model is shown. Bayesian (BA) phylogenetic reconstruction showed a very similar topology. The ML bootstrap values higher than 50 are shown below the branch, and the Bayesian support values over 90% are shown above the branch. Haplotypes found in each population are shown, with population codes corresponding to those listed in Table 3. Sequences corresponding to heterozygous individuals are noted with the polymorphic site in parenthesis.

Prior to the phylogenetic analysis, we collapsed identical haplotypes for each population. Both phylogenetic reconstructions (Maximum Likelihood and Bayesian analysis) had a virtually identical topology and branch support (Figure 5). The ML tree was obtained using the Hasegawa-Kishino-Yano model, the one selected by the inbuilt model generator in MEGA5. It showed *A. sinica* as the most divergent species. The remaining samples were very closely related. The parthenogenetic samples had a total of nine very closely related haplotypes, one of them found in nine populations, was shared with both *Artemia* sp. from Kazakhstan and one of the haplotypes from the Iranian *A. urmiana*, although this latter haplotype contained an indel. The populations of *A. urmiana* from Koyashskoe Lake and *A. tibetiana* present different haplotypes, although still closely related to the parthenogenetic ones.

### Na^+^/K^+^ ATPase

The Na^+^/K^+^ ATPase alignment was 160 bp long and consisted of sequences of 63 individuals. The alignment did not contain indels and had nine polymorphic sites (Table 3). Evidence of heterozygosity was found in all parthenogenetic populations and in only the sexual population from Kazakhstan. The populations from Moimishanskoe Lake (Altai), Gahai Lake (China) and Urmia Lake (Iran) share the same alleles at all polymorphic sites with the sexual population from Kazakhstan (see Table 3).

## Discussion

In order to shed light on the origin and evolution of parthenogenesis in *Artemia*, we explored the genetic variability of nuclear and mitochondrial DNA of diploid parthenogenetic strains and sexual species, with an emphasis on Asia, the region considered to be the most likely centre of origin of asexual lineages [29]–[31]. Our analyses confirmed the existence of at least two and possibly three maternal clades of diversity, two of them most related to two different sexual *Artemia* species, *A. urmiana* and *Artemia* sp. Kazakhstan in agreement with Muñoz et al. [30], but also revealed a possibly new lineage of parthenogenetic lineages represented by KUJ [29]. Overall, nuclear genes indicate that diploid parthenogenetic *Artemia* is very closely related to *A. urmia*na, *Artemia* sp. K*a*z*akhstan* and *A. tibetiana*, with the exclusion of *A. sinica*. Both nuclear and mitochondrial data for *A. sinica* are very divergent to those of diploid parthenogens, suggesting that this species did not contribute to the genetic diversity of diploid parthenogenetic *Artemia*. Our survey substantially expands our knowledge of its genetic diversity in Eurasia.

Our geographically wider number of *Artemia* populations sampled, inclusion of rare males and samples of a recently found population of *A. urmiana* not sequenced before revealed that the lineages in Muñoz et al [45] are not highly supported phylogenetically, as we found further intermediate haplotypes and also identified the key role of the new *A. urmiana* population from Koyashskoe Lake. Furthermore, we found that the less common mitochondrial group (A) is closely related to haplotypes newly sequenced here from *A. urmiana* from Koyashskoe Lake, but occupies a non-monophyletic position in the network between both *A. urmiana* populations, which appears incompatible with a mutational origin, and points to a possible event of contagious parthenogenesis. In contrast, the most common lineage (B), is monophyletic and closely related both to the haplotypes of *Artemia* sp. from Kazakhstan, and to those of two *A. tibetiana* populations from Lagkor Co and Gaize lakes, which represent a new lineage of *A. tibetiana* (see below). Our analysis also revealed a possibly further lineage, so far only found in rare males from Kujalnik population, indicating that they might be present in some populations at low frequencies, maybe resulting from the emergence of new parthenogenetic lineages [29].

In agreement with previous work [30], [38], our results support that the Asiatic sexual species *A. urmiana*, *A. tibetiana* and the undescribed species from Kazakhstan, are closely related such that they might be considered a species complex, despite clear morphological differences [29], [46]. This is further supported by experimental crosses showing that, under laboratory conditions there is a proportion of fertile interspecific crosses between these sexual species, indicating weak post mating isolating barriers to gene flow [25].

*A. tibetiana* contains several divergent, polyphyletic mtDNA lineages, but, in contrast, its nuclear diversity is very homogeneous (monomorphic ITS1 and ATP) and shows little or no differentiation to *A. urmiana* and *Artemia* sp. Kazakhstan. A possibility to explain this pattern is that introgression from other species, in particular from females of *Artemia* sp. Kazakhstan, has resulted on capture of mitochondrial lineages. The genetic diversity of this species needs to be explored further and its taxonomic status might have to be re-evaluated. Given that we have a limited number of samples from *A. tibetiana*, and the richness of hypersaline habitats in Tibet is high [47], [48], it is likely that the level of diversity within *A. tibetiana* might still be underestimated. The mitochondrial lineages of *A. tibetiana* are diverse and the genetic diversity of the rest of the Asiatic species appears to be a subset of it, therefore, *A. tibetiana* might have a key role in the origin of the species complex and the origin of parthenogenetic lineages.

Although mitochondrial markers have allowed us to identify the minimum number of maternal origins of each diploid *Artemia* parthenogenetic lineage, nuclear markers should provide information on both parental species and therefore, shed some light on their modes of origin. For example, diploid parthenogenetic lineages resulting from hybridization between conspecific or interspecific sexuals are expected to have a characteristic signature of high heterozygosity, with diploid asexual lineages containing alleles typical of both parental species [49]. If asexuality arises by contagious parthenogenesis through rare males, we could expect a different maternal origin and possibly distinctive genomic component of parthenogenetic lineages. However, repeated gene flow through contagious parthenogenesis should result in a regular emergence of asexual strains and the genetic differentiation between asexuals and sexuals relatives should be low. Our nuclear analysis shows that ITS-1 from parthenogens is closely related to *Artemia* sp. from Kazakhstan, *A. tibetiana* and *A. urmiana*. Some parthenogens and *Artemia sp*. from Kazakhstan share the same haplotype, whereas *A. sinica* is very divergent. Baxevanis et al. [32] found four parthenogenetic *Artemia* lineages, three of which clustering with *A. urmiana* and *A. tibetiana* and another one more closely related to *A. sinica*. The closely related nature of the sexual species from Asia and the lack of divergence between the investigated nuclear genes, however, make it difficult to assess the mechanism or mechanisms of origin of parthenogenesis. However, our mitochondrial phylogenies do not provide clear evidence of rampant contagious parthenogenesis, as it would result in repeated occurrences of new asexual strains and higher mitochondrial diversity. Moreover, parthenogenetic populations coexisting with the known populations of *A. urmiana* do not have a local origin, as they do not share any haplotypes with the local sexual population. On the contrary, only three mtDNA lineages are found, one of them a minor lineage identified in rare males. That might indicate either that some occasional contagious parthenogenesis does occur or that these are low frequency parthenogenetic lineages with a higher propensity to produce rare males, and have persisted in populations at low frequency. These events would increase the diversity of parthenogenetic strains but playing little role on the geographical expansion and success of parthenogenetic lineages.

The three mtDNA lineages in diploid parthenogenetic *Artemia* are not differentiated in their nuclear DNA. Although this pattern could result both from repeated hybridization between two similar lineages or from a contagious event between one lineage group and another, the possible existence of contagious parthenogenesis is also supported by microsatellite data. The set of microsatellite loci developed for diploid parthenogenetic *Artemia* [45] did not amplify consistently in all the sexual species from Asia [29], [31], suggesting that parthenogenetic strains have enough nuclear distinctiveness, and this may be more consistent with contagious parthenogenesis than with a hybrid origin, although it is possible that different mechanisms underlie the origin of each lineage group.

As we used Manaffar et al.'s [33] primers to amplify and sequence a fragment presumably containing a diagnostic SNP between parthenogenetic and sexual strains, we were able to test their finding on a wider array of samples. Our results indicate that, although most samples from a wide range of parthenogenetic populations do meet this criterion (position 140 in our alignment, see Table 3), we identified some parthenogenetic populations that were homozygous for this position (GAH and MOI) and do not confirm the universality of the polymorphism at this site to distinguish parthenogenetic and sexual populations.

Our data cannot rule out either hybridization between any of the very closely related Asiatic sexual species, or rare events of contagious parthenogenesis via rare males as the contributing mechanisms to the generation of genetic diversity in diploid parthenogenetic *Artemia* lineages. Although our work has provided information on the origin of diploid parthenogenetic *Artemia*, much is still unknown, and the close relationship of sexual species has hampered this, therefore, more research possibly using genomic approaches is needed to disentangle the evolutionary origin of diploid parthenogenetic *Artemia*.
